# Aspartame: Sweetener with anti-inflammatory potential?

**DOI:** 10.4103/0976-500X.72357

**Published:** 2010

**Authors:** Sapna Pradhan, U. H. Shah, A. G. Mathur, S. Sharma

**Affiliations:** *Department of Pharmacology, Army College of Medical Sciences, Near Base Hospital, Delhi, India*

Sir,

Aspartame (L-aspartyl-L-phenylalanine methyl ester) is a nonnutritive intense artificial sweetener. Artificial sweeteners are increasingly being used not only by diabetics, but also by the general public as a means of weight control.

Over 20 years have elapsed since aspartame was approved by the USFDA as a sweetener and flavor enhancer. Several scientific issues continue to be raised after the approval, largely as a concern for theoretic toxicity from its metabolic components the amino acids, aspartate and phenylalanine and methanol even though dietary exposure to these components is much greater than from aspartame.[1] A safety review concluded that the acceptable daily intake of 40 mg/kg/day of aspartame is entirely safe except for phenylketonurics.[2] Current available literature suggests that the earlier safety concerns have not been substantiated.[3–9]

Allen B Edmundson, an X-ray crystallographer with diagnosed generalized osteoarthritis, noted unexpected relief of arthritic pain and joint stiffness after he consumed 6 glasses of diet coke containing approximately 1.1 gm aspartame while watching a football match. Subsequent investigations revealed that aspartame appears to relieve pain, induce mild antithrombotic effects in humans and decrease fever in animals.[10]

This observational report prompted us to review the literature on the biological effects of aspartame. Since there were only two studies reporting the anti-inflamatory effects of aspartame, it was considered worthwhile to conduct the present study. “Inhibition of formalin-induced ascites in mice” was selected as the experimental model since anti-inflamatory effects have not been studied in this model so far.

Albino Wistar rats of either sex, weighing 250 g were selected for the study. They were caged individually under controlled conditions with 12 h dark and light cycles and had access to food and water *ad libitum*. All the experiments were carried out at the same time of the day. The care and maintenance of animals were as per the approved guidelines of the Committee for the Purpose of Control and Supervision of Experiments on Animals, India. The study was carried out after obtaining permission of Institutional Animal Ethics Committee of B J Medical College, Ahmedabad. Aspartame was obtained from Zydus Cadila, Ahmedabad.

Fasting animals were administered either vehicle (distilled water) or aspartame dissolved in 0.2 ml distilled water intraperitoneally. An hour later, all animals received 1 ml of 1.5% formalin solution intraperitoneally by a 26-gauge needle. After further starvation for 6 h, the animals were sacrificed by cervical dislocation. The abdominal cavity was opened and collecting funnel used to empty peritoneal contents into numbered test tubes.

The animals were randomly divided into 5 groups of 10 animals each. Animals in Group I (control) received 0.2 ml distilled water. Animals in groups II, III and IV received aspartame in doses of 2, 4 and 8 mg/kg, respectively. Animals in Group V were administered diclofenac 1 mg/kg.

The anti-inflammatory activity is indicated by the decrease in the quantity of ascitic fluid collected in drug-treated animals and by “percentage protection,” which is calculated by using the formula:

$$\%\;\mathrm{protection}\;=\;100\;-\;\left\{\frac{\mathrm{amount}\;\mathrm{of}\;\mathrm{fluid}\;\mathrm{in}\;\mathrm{drug}\;\mathrm{treated}\;\mathrm{group}}{\mathrm{amount}\;\mathrm{of}\;\mathrm{fluid}\;\mathrm{in}\;\mathrm{control}\;\mathrm{group}}\;\times \;100\right\}$$

Statistical analysis was performed by applying Student’s unpaired *t* test.

Table 1 shows the effect of aspartame and diclofenac on formalin-induced ascites in rats. At the doses of 4 and 8 mg/kg, aspartame showed a statistically significant (*P* < 0.01) anti-inflammatory activity (24.41% and 31.02% inhibition, respectively). These results are comparable to the effects of diclofenac, a standard anti-inflammatory agent (36.90% inhibition).

**Table 1:** Effects of aspartame and diclofenac on formalin-induced ascites in rats

Group	Vol. of ascitic fluid (ml)	% Protection
Group I: 0.2 ml distilled water (control)	1.87 ± 0.15	--
Group II: aspartame 2 mg/kg	1.42 ± 0.11	24.06
Group III: aspartame 4 mg/kg	1.32 ± 0.12*	24.41
Group IV: aspartame 8 mg/kg	1.29 ± 0.10*	31.02
Group V: diclofenac 1 mg/kg	1.18 ± 0.10*	36.90

Values are mean ± SEM; n =10 in each group.

* *P*<0.01 as compared to control

A review of the available literature on the subject revealed only two studies evaluating the anti-inflammatory effect of aspartame. In one study which investigated analgesic and anti-inflammatory properties of aspartame alone as well as in combination with various opioids and nonsteroidal anti-inflammatory drugs, it was found that aspartame in the doses of 4, 8 and 16 mg/kg, p.o. showed a significant response in the carrageenan-induced rat paw edema model.[11] The present study corroborates the role of aspartame as an anti-inflammatory agent. This correlation is further strengthened by the fact that our study was performed in a different model in which it has not been studied earlier.

The second study involved chronic administration of aspartame for 6 days followed by induction of carrageenan-induced monoarthritis.[12] Interestingly, although aspartame in a dose of 50 mg/kg reduced mechanical pain, it did not demonstrate any significant anti-inflammatory effect. These findings appear to contradict the anti-inflammatory effect of aspartame in doses of 4 and 8 mg/kg observed in our study.

Although aspartame has demonstrated anti-inflammatory potential in two separate experimental models of inflammation in soft tissues, it has failed to display the same in experimental arthritis despite the increased dose and duration. One possible explanation of these findings could be the differences in the underlying pathophysiologic mechanisms of inflammation in the three different experimental models. It is suggested that anti-inflammatory effect of aspartame could be further evaluated in other animal experimental models.

In view of the above studies and its reported analgesic and antipyretic effects, aspartame holds promise as a potential therapeutic agent. Further pharmacological studies including clinical studies are required to explore the full therapeutic potential of aspartame.
